# Contact Zone of Asian and European Wild Boar at North West of Iran

**DOI:** 10.1371/journal.pone.0159499

**Published:** 2016-07-21

**Authors:** Parinaz Khalilzadeh, Hamid Reza Rezaei, Davoud Fadakar, Malihe Serati, Mansour Aliabadian, James Haile, Hamid Goshtasb

**Affiliations:** 1 Department of Fishery and Environment, Gorgan University of Agricultural Science and Natural Resources, Gorgan, Golestan, Iran; 2 Department of Natural Resources, Isfahan University of Technology, Isfahan, Isfahan, Iran; 3 Department of Biology, Faculty of Sciences, Ferdowsi University of Mashhad, Mashhad, Khorasan Razavi, Iran; 4 PalaeoBARN, Research Laboratory for Archaeology and History of Art, Institute of Archaeology, Oxford University, Oxford, United Kingdom; 5 Department of Environmental Sciences, College of Environment, Karaj, Alborz, Iran; University of York, UNITED KINGDOM

## Abstract

Wild boar (*Sus scrofa*) are widely distributed throughout the Old World. Most studies have focused on Europe and East Asia with the genetic diversity of West Asia being less well studied. In particular, the genetic variability and genetic structure of the Iranian populations are not yet known; gaps which prevent scientists from resolving the genetic relationships of the Eurasian wild boar. This paper is the first attempt to provide information about genetic relationships among modern Iranian populations of the Eurasian wild boar (*S*. *scrofa*) by sequencing 572 bp of the mitochondrial (mt) DNA control region. As a result of this investigation, it was discovered that Iran contains not only Middle Eastern haplotypes, but also shares haplotypes with Europe and East Asia. The Italian clade, which is endemic in Italy, is not identified in Iran, while all other clades, including Asiatic, European, Near East 1, and Near East 2 are found based on the phylogenetic tree and median-joining network. The results of this study illustrate that north west of Iran (specifically Southwest Caspian Sea) is the contact zone between the Asian (Near Eastern and Far Eastern), and the European clades. In light of the fact that the domestication of pigs occurs in Anatolia, this finding is important.

## Introduction

Wild boar (*Sus scrofa*), one of the world’s most widely distributed species, is a native to Europe, North Africa, and Asia, and have also been artificially introduced to other continents with the exception of Antarctica [1,2]. Iranian wild boar populations, which inhabit the wide geographical range of the country [3], have increased recently due to their high rate of reproduction, lack of hunting because eating their meat is forbidden (*haram*) in Islam, and decrease in their natural predators, such as wolf (*Canis lupus*) and Persian leopard (*Panthera pardus*); hence, wild boar is considered as a pest in many parts of Iran because they damage agriculture crops [4].

The evolutionary history and genetics of wild boar have received much attention [1,2,5–15] because of the animals’ importance in ecological and economic roles in human life and natural ecosystems [16]. Phylogenetic relationships and genetic diversity of wild boar are well understood in Europe and East Asia [15,17,18] and the results indicate that pigs may be derived from two maternal origins: Asian and European wild boars.

Five major clades are identified for Eurasian wild boar consisting of Asiatic, Near Eastern1 (NE1), Near Eastern 2 (NE2), European and Italian [19,20]. The Asiatic clade is widespread in East Asia, while NE1 and NE2 are widespread in West Asia with sympatric distribution in the Near East. European clade is distributed through Europe, while the Italian clade is restricted to Italy. Iberian wild boars with a high proportion of unique haplotypes [21], Spanish wild boars [21], and Brazilian wild boars [22] all belong to the European clade [6]. Genetic divergence between European and East Asian wild populations and domestic breeds is clear [1,5,9,23,24], while the contact zone of Asian and European wild boars is yet unknown [1].

Genetic diversity and structure of wild boar in Central and Eastern Europe are relatively weak [25]; however, high level of genetic variation is found in South-Western Europe. A recent study demonstrated high levels and diverse patterns of genetic variation among regional populations of wild boar from East Asia. This diversity decreases gradually from Southeastern Asia to Northeastern Asia [2].

Unfortunately, despite the extensive studies, information about the genetics of Iranian wild boars are mainly limited to the studies of Ottoni et al. [20] and Larson et al. [10,19]; in other words, Iranian wild boar phylogenetic still remains challenging. It is obvious that animal mitochondrial DNA (mtDNA) is a useful marker [26,27] which has been frequently used to estimate the genetic diversity and relationships between closely related species and within species, such as wild boar across Eurasia [15,20,28–30]. mtDNA is highly polymorphic, almost exclusively maternally inherited and without genetic recombination [21,31,32]; therefore, the mtDNA control region can be applied to clarify the phylogeny and genetic variation of wild boar in Iran.

This paper focuses on the genetic relationships of wild boar populations of Iran in order to understand their phylogenetic relationships and fill gaps in the description of wild boar mtDNA variation.

## Materials and Methods

### Sampling

Fresh muscle or skin fragments were sampled from 54 wild boar in Iran (S1 Fig) shot for the purpose of wild boar management with the permission of and in accordance with the national regulations of the Iranian Department of Environment. Samples were either stored in plastic tubes (5 to 30 ml) filled with 96% alcohol or kept frozen at -20°C. As the animals were not killed for the primary purpose of obtaining samples, an ethics statement was not required and as access to sampling locations was not restricted, no specific permissions were required. The locality information, voucher numbers and accession numbers are summarized in S1 Table.

### DNA extraction, amplification and sequencing

Whole genomic DNA was extracted from tissue samples using AccuPrep genomic DNA extraction tissue kit (Bioneer) following the manufactures’ instructions. Polymerase chain reaction (PCR) was performed for amplification of 572 base pairs (bp) fragment of the control region of mtDNA using L15387 (5`-CTCCGCCATCAGCACCCAAAG-3`) and H16108 (5`-GCACCTTGTTTGGATTRTCG-3`) primers [10]. The reaction mixture was prepared in 25-μl volumes, containing 1 unit of Euro Taq DNA polymerase, 10 μM Tris-HCl, 30 μM KCl, 1.5 mM MgCl_2_, 250 μM of each dNTP and 2 pmol primer (Bioneer, South Korea). Thermocycling was performed as follows: 3 min at 94°C followed by 30 cycles of 45 s at 94°C, 45 s at 60°C, and 50 s at 72°C, and finally followed by 5 min at 72°C. Double-strand cycle Sanger sequencing was performed using the Big Dye Terminator Cycle Sequencing kit version 3.1 (Applied BioSystems) and electrophoresis of the purified sequencing product was carried out on an ABI PRISM 3730xl automatic sequencer.

### Data analysis

Sequences were edited with SeqScape version 2.6 software (Applied Biosystems). All new sequences have been submitted to GenBank (accession numbers KR075765—KR075818). All datasets were aligned using the Clustal W algorithm [33] implemented in Mega5 [34], and final adjustments were performed by eye. In addition, a sequence of *Phacochoerus africanus* (NC008830) [30] was chosen as an outgroup (S1 Table). The most appropriate model of nucleotide change was selected using jModeltest 0.1.1 [35]. The best model was the HKY model [36], according to both the Akaike information criterion (AIC) and the Bayesian information criterion (BIC), with gamma-distributed (G) rate variation across sites. Bayesian phylogenetic analyses were carried out in MrBayes v3.2 [37] using the HKY+G model of sequence evolution and two independent runs of four Markov chains (one cold and three heated) over 10,000,000 generations and sampling every 100 generations. The first 25% of the sampling trees and estimated parameters were discarded as burn-in. Convergence was monitored by the decrease in standard deviation of split frequencies and the potential scale reduction factor (PSRF) associated with the model parameters.

Population genetic structure was estimated among and within populations by an analysis of molecular variance (AMOVA) and Fst statistics were calculated using Arlequin software v3.0 [38]. AMOVA of populations are done following the geographic provinces and the province/geographic locations reflect the ID-name of the specimens in S1 Table and S1 Fig.

Based on a 572-bp fragment of the mtDNA control region, a median-joining (MJ) network was constructed using all wild haplotypes obtained in the present study using NETWORK v4.1.0 [39]. It shows the Iranian clades and haplotypes in further detail, aiming to clarify the relationships between the haplotypes described in the present study. A second MJ network was created with the same procedure, using our data plus 36 published sequences.

## Results

In total, 54 specimens were successfully amplified and aligned. The sequences in our data set were then aligned with 36 *S*. *scrofa* sequences available from GenBank. A complete list of sequences and corresponding haplotypes used in the present study is shown in S1 Table. AMOVA analysis showed a significant genetic differentiation among wild boar populations (Fst = 0.86; *P* < 0.0001) (Table 1).

**Table 1:** Analysis of molecular variance Analyze.

Source of variation	d.f.	Variance of components	Percentage of variation	*P*
Among populations	9	98.58	86.21	< 0.0001
Within populations	44	15.76	13.79	
Total	53	114.34		
Fixation Index Fst	0.86213			

**Fig 1:**
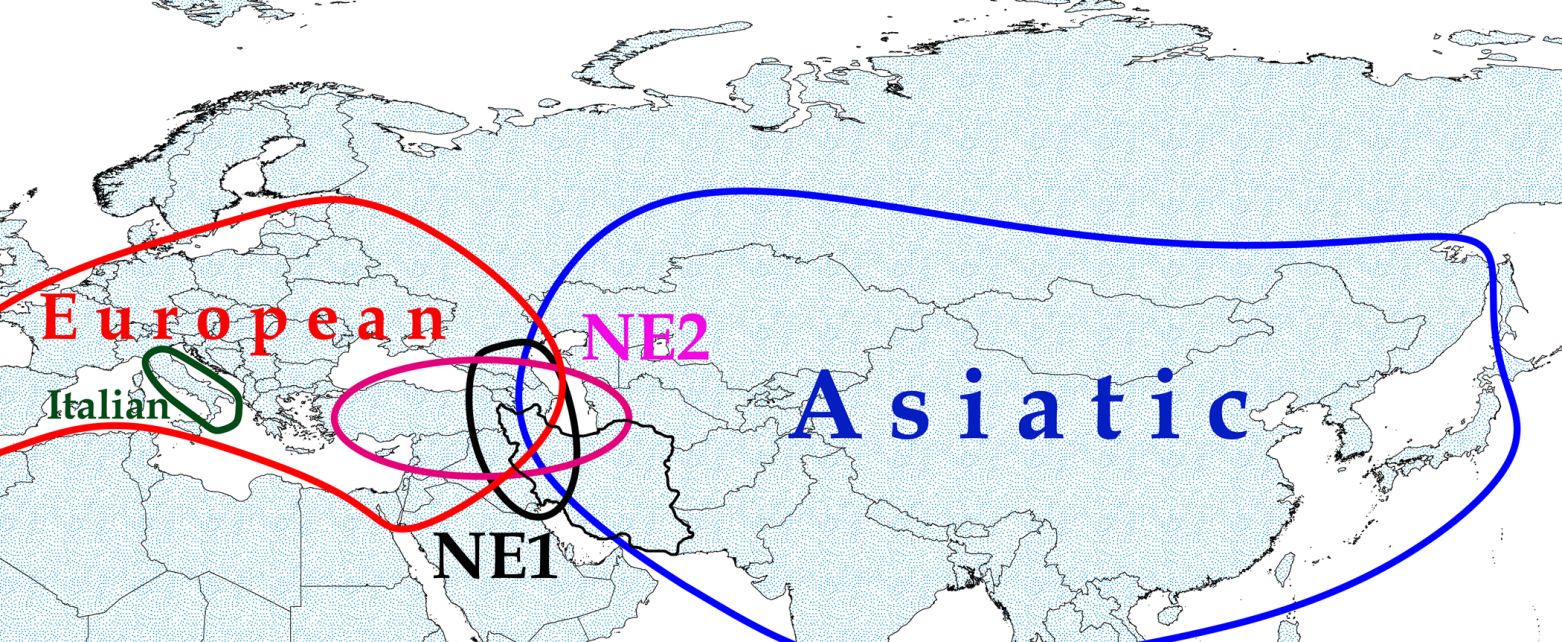
Geographical distribution of wild boar clades in the Old World. The shapefile of global country boundaries was downloaded from DIVA-GIS dataset and the layout was made in QGIS version 2.4. Original copyright [2016].

**Fig 2:**
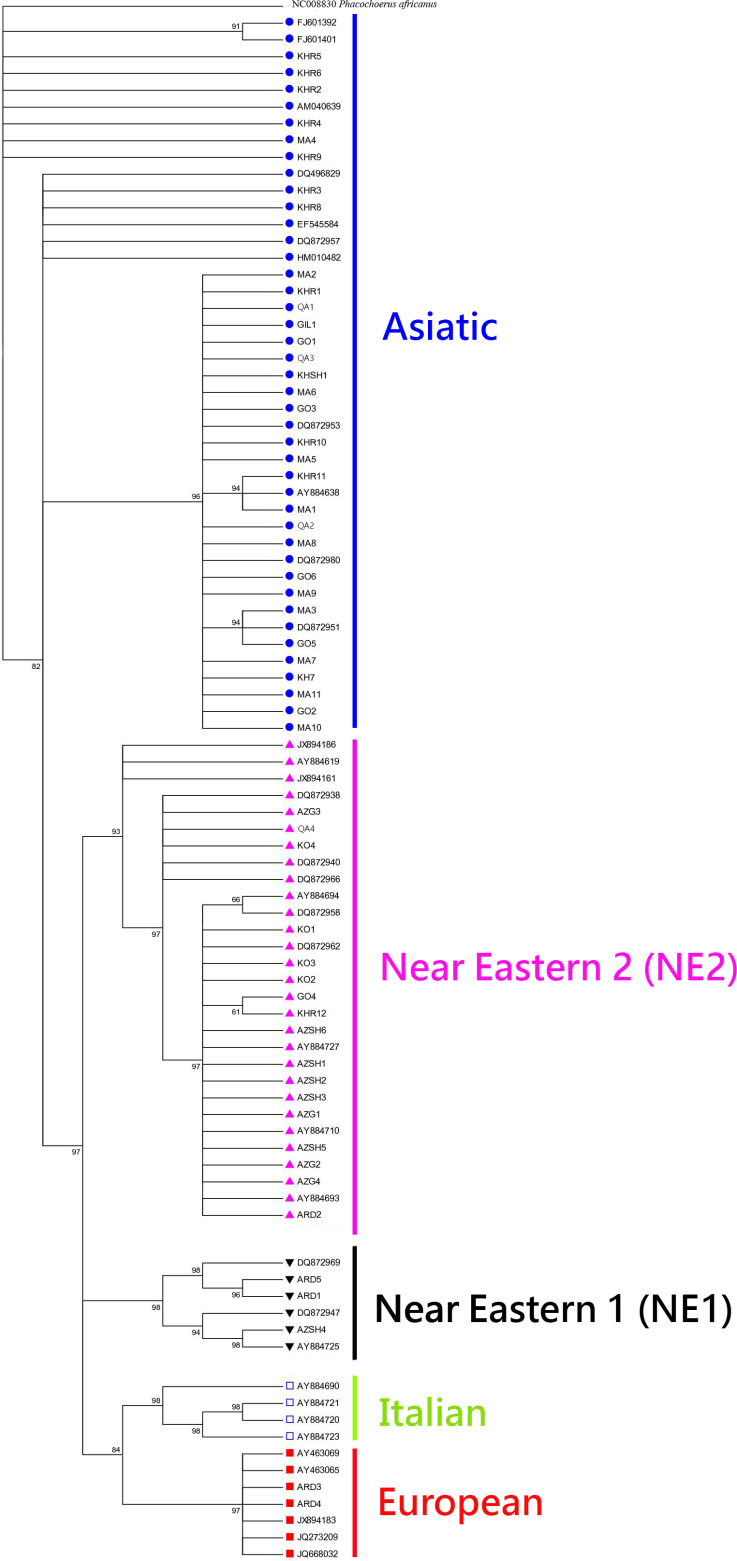
Phylogeny based on the alignment of the sequences of control region gene. Bayesian analysis was performed with 90 sequences with the HKY + G substitution model and numbers on nodes depict Bayesian posterior probabilities. For sequences obtained from GenBank accession numbers are given.

Phylogenetic analyses clearly separate five clades consisting of Asiatic, Near Eastern 1 (NE1), Near Eastern 2 (NE2), European and Italian. These five clades are geographically distributed in the Old World (Fig 1). The two predominate clades in the Near East are NE1 and NE2. The European clade is a major feature of European diversity beside the Italian clade distributed in Europe, and Asiatic clade encountered as a most frequent clade in Asia from the Far East to Near East (Fig 2). The tree shows the position of the wild boar of Iran within other wild boars of the Old World. Surprisingly, the sequences of Ardabil province (ARD3 and ARD4) are clustered within the European clade. The Asiatic (59.2%) and NE2 (31.4%) are the most frequent clades in Iran, respectively (NE1 clade 5.5%, European clade 3.7%).

**Fig 3:**
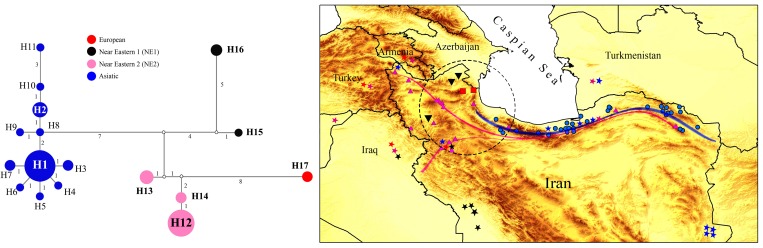
Median-joining (MJ) network of mitochondrial control region haplotypes (572-bp) observed in 54 wild boars (*S*. *scrofa*) from Iran. Circle size is proportional to haplotype frequencies; filling color refers to the main clades for the wild boars of Iran (Asiatic, Near Eastern 2, Near Eastern 1, and European). Mutational steps among haplotypes are signaled with the number of mutations and small, filled white, circles refer to inferred missing haplotypes. The map shows the point-based distribution of samples of wild boar of Iran which is used to make Bayesian phylogenetic tree and MJ network. The colors of points refer to clades in the tree and MJ and stars on the map indicate samples from other studies in Iran, and also clades that distribute in other countries. The background hillshade was made using the Shuttle Radar Topography Mission (SRTM) elevation model (http://srtm.csi.cgiar.org) in QGIS version 2.4. Original copyright [2016].

An MJ network has been constructed in order to overview haplotype distribution and relationships in wild boars from Iran (Fig 3). Fig 3. demonstrates the relationships among haplotypes which consists of 17 haplotypes. The eight haplotypes (H4-6, H8-11, and H15) were represented by a single sequence, while all others were found in at least two specimens. Of the 17 haplotypes observed in Iranian *S*. *scrofa*, H1 (17 individuals) and H12 (12 individuals), which belong to Asiatic and NE2 clades, respectively have the highest frequency and the widest geographical distribution. Of the haplotypes classified in the NE1 clade, H16 (ARD1 and ARD5) is the most frequent and is found in Ardabil province at Northwest Iran (38 15′ 0″ N, 48 17′0″ E). The Asiatic clade is present in an East-West gradient in Iran, and H1 as core haplotype was surrounded by a star-like pattern. European clade is separated from NE2 by eight mutations. In Iran, this clade is only observed in two wild boar specimens from the Southwest Caspian Sea which belong to the H17 haplotype. H1 as a major one includes GA1, 2, 3; GI1; MA5, 6, 7, 9, 10, 11; GO1, 2, 3, 6; KHR7, 10; and KHSH1. Specimens from Mazandaran province have a large proportion of the H1. In MJ network, it is possible to identify four distinct clades (Asiatic, Near Eastern 2, Near Eastern 1, and European).

**Fig 4:**
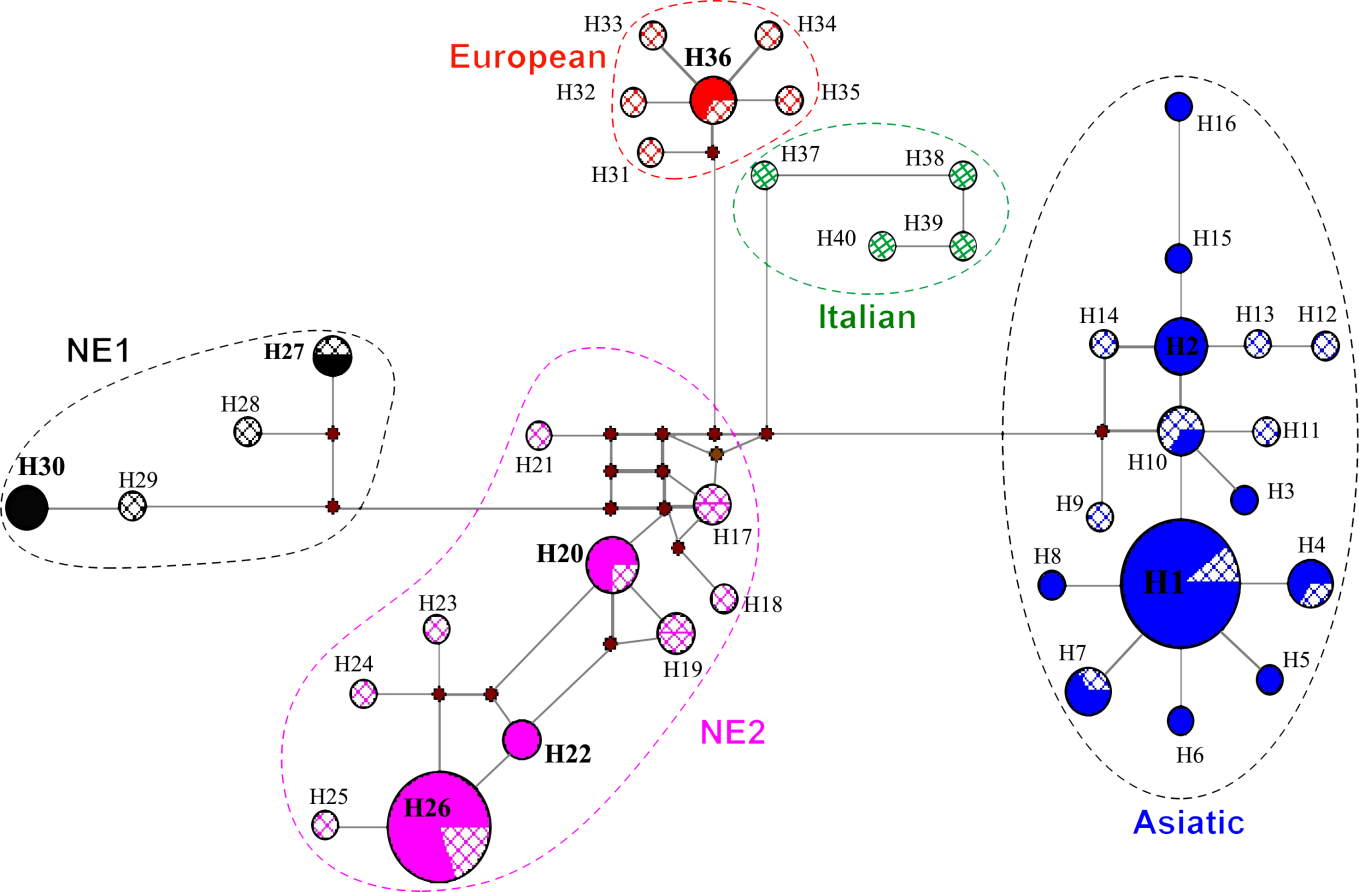
Median-joining network based on a 572-bp fragment of mtDNA control region depicting the relationships among the main five clades described for wild boars (Asiatic, Near Eastern 2, Near Eastern 1, European, and Italian), delimited by dashed lines. The haplotype shown in diagonal cross corresponds to sequences from GenBank, and solid with color ones are from Iran. Additional details about clades, haplotypes, and accession numbers are provided in S1 Table. Black dots showing missing haplotype and circle sizes are proportional to haplotype frequencies and number refer to haplotype codes.

An MJ network based on a 572-bp fragment of the mtDNA control region and constructed with all sequences of present study plus 36 published sequences (S1 Table), is represented in Fig 4. This median-joining haplotype network showed a star-like radiation with five distinct clades (Asiatic, Near Eastern 2, Near Eastern 1, European, and Italian).

## Discussion

Existing studies revealed genetic relationships among wild boar populations in the Old World although insufficient sampling left a large gap in Western Asia, especially in Iran. This gap prevented the identification of possible ‘suture zones’, or support for any hypotheses of genetic variation of wild boar in Eurasia [1]. This study is the first to present a comprehensive dataset on the genetic structure of the wild boar populations in Iran. Our results show that wild boar populations share a European mitochondrial genetic signature in the Northwest Iran (Fig 2). The most striking result to emerge from the data is that the contact zone of the Asian distribution (i.e. NE1, NE2, and Asiatic) and the European clade is in the Southwest Caspian Sea.

In Iran, wild boar are an ecological pest, and control activities are used to eradicate or limit the populations. Yet our data showed high levels of gene diversity in wild boar populations of Iran. The phylogenetic tree of mtDNA control region showed five clades and is in agreement with previous studies of *S*. *scrofa* [10,19,20].

According to the Bayesian phylogenetic tree (Fig 2), Iran is populated by wild boar belonging to four major clades: Asiatic, Near Eastern 1, Near Eastern 2, and European clades. The Asiatic clade is widespread throughout the east of the country, whereas NE2 clade is widespread in the west. The Asiatic clade is not only the most widespread in Eastern Iran, but also the most diverse, with two widely distributed clusters (Fig 3): East Iran-side of the Asiatic clade (H8-11 and H2), which is common in the Khorasan province in Northeast Iran, but is rare in the Caspian Sea region in North Iran; and Caspian Sea side of the Asiatic clade (H3-7 and H1), which is widespread in the east, and also throughout the Caspian Sea region in North and Northeast Iran. On the other hand, an east-to-west gradient of genetic variation seems to exists.

Asiatic and NE2 clades are more frequent than two other clades. The distribution range of Asiatic clade is from Khorasan province in the East to Qazvin province in the West, while European clade is restricted to the Ardabil province in the Southwest Caspian Sea. Furthermore, Near East 2 is more frequent than Near East 1, and overlay each other in West Iran (Fig 3). Asiatic and NE 2, as two major clades of the wild boar of Iran, overlay each other in the Caspian Sea region.

European pigs were brought to the Levant by the Sea Peoples who migrated there [40]. In Iran, the European clade is the minor one and consists of individuals from Ardabil province in Northwest Iran, while some European specimens have been reported by Larson et al. [19] from Near East, including one specimen from Kermanshah province in West Iran (DQ872939) and two specimens from Iraq (DQ872959 and DQ872946) (Fig 3). Ardabil is the easternmost distribution the European clade, a clade that is widespread in nearby Armenia [20,21]. ARD3, ARD4, and European specimens (AY463069, AY463065, JQ273209, JQ668032, and JX894183) are in the European clade (Fig 2) which are the same haplotype as JX894183 in the MJ network (Fig 4). The ancient DNA data indicated that European clades are not distributed in the Middle East and recent studies [20,40] propose translocations from Europe to the Middle East as an explanation for this structure. The process of human-mediated dispersal of European domestic pigs followed by feralization might explain the current European clade present in Iranian wild boar. Therefore, two pathways could be recognized if the European clade is connected from east to west, from Ardabil in Northwest Iran to Armenia and then to North Black Sea to Europe; and from Ardabil to Kermanshah province in West Iran to Iraq, Syria, and Turkey.

The results obtained by the AMOVA analysis show that most of the variation is observed among populations, which is a strong indication of their genetic structure. It is also possible to recognize that a few haplotypes are exclusive to particular geographic locations (Fig 3), providing evidence for this structure and for the uniqueness of some populations. Taken together with the high levels of haplotype variation observed in the contact zone of the Asian (NE1, NE2, and Asiatic) and European clade in the Caspian Sea region, this may reflect the existence of glacial refugia for *S*. *scrofa* in the Caspian Sea region during the last glacial [41]. On the other hand, haplotypes, such as H1, H2, and H12 are widely distributed, which suggests that these are ancestral haplotypes or alternatively, these haplotypes result from expanding populations, and also assuming that the ancestral haplotypes are internal while the derived haplotypes are peripheral. H1 with six connections to other haplotypes, and H8 with four connections, are relatively ancestral compared to the remaining haplotypes.

In the MJ network of control region sequences, Asiatic and Near East 2 clades show a broad geographical distribution, which suggests these might be the oldest because of their high frequency throughout the sampling areas. The NE2, which is more frequent than NE1 clade, is connected with the European clade in the MJ network and this result is consistent with Larson et al. [19] and Ottoni et al. [20] who indicate that a different distribution of NE1 (which does not include the alleged centers of domestication in Southeast Anatolia) and NE2 (which on the other hand includes the centers of domestication) is in line with the fact that only haplotypes belonging to clade NE2 were involved in the domestication process (and transported to Europe), whereas NE1 was left out. Therefore, the contact zone between NE2 and European clades is important to address the questions about the dispersal of NE2 in Europe because the NE2 is widespread in West Iran and Anatolia, and also the detail of the distribution of the NE2 and its relationships with other clades might provide deeper insight into domestication process.

Furthermore; the MJ network obviously shows that the Iranian wild boar do not belong to a unique clade. Haplotypes H1 and H12 correspond to the two core lineages of Asiatic and NE2 clades that occur at high frequency in Iran; therefore, Asiatic and NE2 clades are widely distributed in Iran. H1 was surrounded by a star-like pattern that could be the result of a recent population expansion. Also, the Italian clade as a second clade of Europe is not distributed in the Middle East and it is geographically limited in Europe. In a study based on ancient DNA, Larson et al. [19] revealed that the Italian clade was only found on the Italian peninsula and Croatia in the past even before the Neolithic, and this clade was not found in Iran. These findings corroborate with studies carried out in the Old World which indicate that the Italian clade is restricted to Europe [10,19,20]. Three main groups, including the Italian, European, and Asian clades were revealed in the analysis of mtDNA sequence variations of Italian wild boar populations [18] and this finding suggested that interbreeding has probably occurred between the wild and domesticated strains in Italy.

Fst is a standard parameter which provides an estimate of the genetic differentiation among and within populations [42,43]. As shown in Table 1, Iran has a high genetic diversity. Different aspects of the ecological and demographic history of the species affect the rate of Fst. Also, genetic admixture is a key factor in genetic diversity and it can be affected by different variables, such as population size, initial genetic diversity, local adaptation and speciation. In line with the fact that immigration is one of the effective factors in the gene flow [44], probably geographical barriers or man-made barriers, such as roads, prevent migration and connection among populations and might reduce genetic diversity, especially in West Azerbaijan and East Azerbaijan provinces.

In general, haplotype variation is relatively high in Northwest Iran. Constructed phylogenetic tree (Fig 2) and MJ network (Fig 3) demonstrate the existence of the hotspot of genetic diversity in this region. This high mtDNA haplotype variation in Northwest Iran is due to introgression from domestic pigs (which notoriously carry European and Asian haplotypes). Therefore, the contact zone should be considered in protection programs. Future genomic analysis will certainly shed more light on the conservation of diversity which should be considered in conservation and management programs of wild boar and in measures to limit its habitat destruction through controlled hunting.

The Northwest Iran represents a hotspot of genetic diversity, hosting all Eurasian mtDNA clades except the Italian clade. Our research underlines the importance of Iranian wild boar populations and the evidence from this study points towards the idea that the Near East is an important area to address questions about genetic relationships between the Asian and European clades. Despite the fact that there are limitations due to small sample size, our work is be a starting point for revealing genetic relationship between Asia and Europe and begins to fill the gaps. The present study provides new insights into the genetic diversity of Iranian wild boar populations, and their relationships with other Eurasian pigs. It is also the first step towards enhancing our understanding of the origins and structure of Iranian wild boar populations. The distribution of the European clade is from Europe to the Levant [40], Armenia [10,20] and Iran; the Asiatic clade is distributed from the Far East to the Middle East [10,19,30,40,32]; and NE1 and NE2 are widespread in East Asia [10,19,20]. These four clades are connected to each other in Northwest Iran and they overlap in Southwest Caspian Sea in Ardabil, Mazandaran, Qazvin and Gilan provinces. The present findings might help to answer questions about gene flow and also Eurasian pig domestication in particular in the apparent overlapping zone. Future work will concentrate on this aspect.

## Supporting Information

S1 Fig The locations of wild boar samples from Iran. The code for each sample is specific and additional details are provided in S1 Table. The country boundaries were downloaded from DIVA-GIS dataset (http://www.diva-gis.org/Data) and the layout was made in QGIS version 2.4. Original copyright [2016].
(TIF)

S1 Table List of the wild boar and domestic pig mtDNA sequences used in this study downloaded from GenBank or obtained by authors of this study. (DOCX)
