# Supplementary figures and images for: Contact Zone of Asian and European Wild Boar at North West of Iran
Source: PLoS One. 2016 Jul 21;11(7):e0159499. doi: 10.1371/journal.pone.0159499 (PMC4956230; doi:10.1371/journal.pone.0159499)

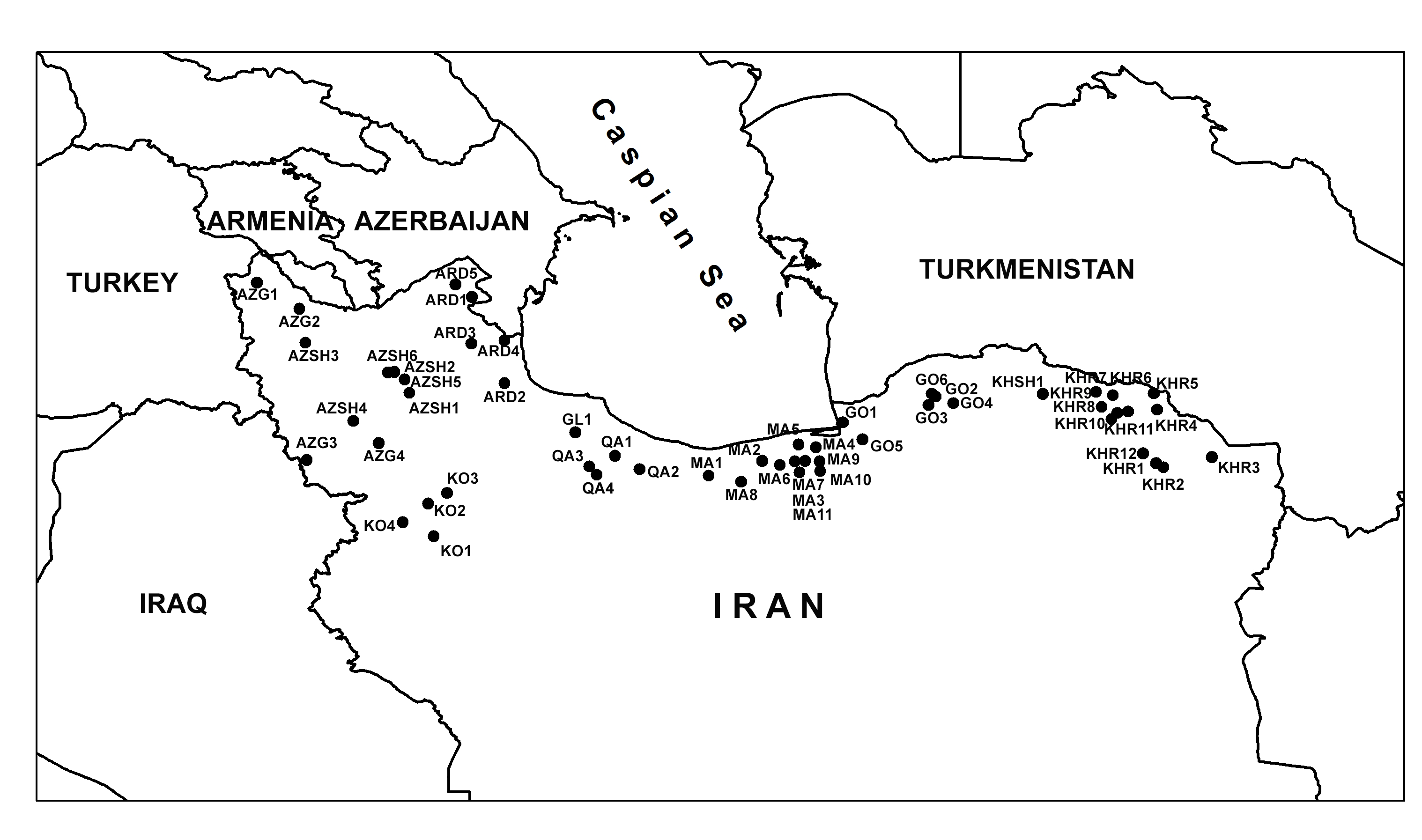

Supplement: S1 Fig — The code for each sample is specific and additional details are provided in S1 Table. The country boundaries were downloaded from DIVA-GIS dataset (http://www.diva-gis.org/Data) and the layout was made in QGIS version 2.4. Original copyright [2016]. (TIF) [file pone.0159499.s001.tif]
