# Supplementary material for: Contact Zone of Asian and European Wild Boar at North West of Iran
Source: PLoS One. 2016 Jul 21;11(7):e0159499. doi: 10.1371/journal.pone.0159499 (PMC4956230; doi:10.1371/journal.pone.0159499)
Supplement: S1 Table — (DOCX) [file pone.0159499.s002.docx]

S1 Caption

**S1 Fig. The locations of wild boar samples from Iran.** The code for each sample is specific and additional details are provided in S1 Table. The country boundaries were downloaded from DIVA-GIS dataset and the layout was made in QGIS version 2.4.

**S1 Table. List of the wild boar and domestic pig mtDNA sequences used in this study downloaded from GenBank or obtained by the authors of this study**

S1 Table.

| **Accession No.** | **Species** | **ID No.** | **Country** | **Location** | **Longitude** | **Latitude** | **Status** | **Reference** |
| --- | --- | --- | --- | --- | --- | --- | --- | --- |
| **KR075783** | ***S. scrofa*** | **KO1** | **Iran** | **Kurdistan- Hasanabad** | **47.657** | **35.855** |  | **This study** |
| **KR075784** | ***S. scrofa*** | **KO2** | **Iran** | **Kurdistan- Hasanabad** | **47.656** | **35.854** |  | **This study** |
| **KR075782** | ***S. scrofa*** | **KO3** | **Iran** | **Kurdistan- Bijar** | **47.628** | **35.890** |  | **This study** |
| **KR075779** | ***S. scrofa*** | **KO4** | **Iran** | **Kurdistan- Sanandaj** | **46.939** | **35.386** |  | **This study** |
| **KR075788** | ***S. scrofa*** | **GL1** | **Iran** | **Gilan** | **49.738** | **36.845** |  | **This study** |
| **KR075776** | ***S. scrofa*** | **AZG1** | **Iran** | **Azerbaijan Garbi- Maku** | **44.561** | **39.277** |  | **This study** |
| **KR075777** | ***S. scrofa*** | **AZG2** | **Iran** | **Azerbaijan Garbi- Chaldiran** | **45.256** | **38.852** |  | **This study** |
| **KR075778** | ***S. scrofa*** | **AZG3** | **Iran** | **Azerbaijan Garbi- Mirabad** | **45.378** | **36.396** |  | **This study** |
| **KR075781** | ***S. scrofa*** | **AZG4** | **Iran** | **Azerbaijan Garbi- Shahin Dej** | **46.546** | **36.673** |  | **This study** |
| **KR075771** | ***S. scrofa*** | **AZSH1** | **Iran** | **Azerbaijan Shargi- Hashtrood** | **47.048** | **37.487** |  | **This study** |
| **KR075772** | ***S. scrofa*** | **AZSH2** | **Iran** | **Azerbaijan Shargi- Bostanabad** | **46.973** | **37.706** |  | **This study** |
| **KR075773** | ***S. scrofa*** | **AZSH3** | **Iran** | **Azerbaijan Shargi- Tasuj** | **45.359** | **38.295** |  | **This study** |
| **KR075768** | ***S. scrofa*** | **AZSH4** | **Iran** | **Azerbaijan Shargi- Miandoab** | **46.132** | **37.025** |  | **This study** |
| **KR075774** | ***S. scrofa*** | **AZSH5** | **Iran** | **Azerbaijan Shargi- Bostanabad** | **46.804** | **37.829** |  | **This study** |
| **KR075775** | ***S. scrofa*** | **AZSH6** | **Iran** | **Azerbaijan Shargi- Diznab** | **46.695** | **37.821** |  | **This study** |
| **KR075785** | ***S. scrofa*** | **QA1** | **Iran** | **Qazvin** | **50.091** | **36.152** |  | **This study** |
| **KR075780** | ***S. scrofa*** | **QA4** | **Iran** | **Qazvin- Alamout** | **50.388** | **36.462** |  | **This study** |
| **KR075786** | ***S. scrofa*** | **QA2** | **Iran** | **Qazvin** | **50.781** | **36.246** |  | **This study** |
| **KR075787** | ***S. scrofa*** | **QA3** | **Iran** | **Qazvin** | **49.967** | **36.292** |  | **This study** |
| **KR075767** | ***S. scrofa*** | **ARD1** | **Iran** | **Ardabil- Keshtosanate Mogan** | **47.878** | **39.664** |  | **This study** |
| **KR075765** | ***S. scrofa*** | **ARD2** | **Iran** | **Ardabil- Khalkhal** | **48.589** | **37.646** |  | **This study** |
| **KR075769** | ***S. scrofa*** | **ARD3** | **Iran** | **Ardabil- Nemin** | **48.050** | **38.285** |  | **This study** |
| **KR075770** | ***S. scrofa*** | **ARD4** | **Iran** | **Ardabil- Sabalan** | **48.593** | **38.336** |  | **This study** |
| **KR075766** | ***S. scrofa*** | **ARD5** | **Iran** | **Ardabil- Parsabad** | **47.855** | **39.662** |  | **This study** |
| **KR075789** | ***S. scrofa*** | **MA1** | **Iran** | **Mazandaran- Kiasar** | **53.644** | **36.205** |  | **This study** |
| **KR075790** | ***S. scrofa*** | **MA2** | **Iran** | **Mazandaran- Kiasar** | **53.551** | **36.241** |  | **This study** |
| **KR075791** | ***S. scrofa*** | **MA3** | **Iran** | **Mazandaran-Kiasar** | **53.711** | **36.374** |  | **This study** |
| **KR075792** | ***S. scrofa*** | **MA4** | **Iran** | **Mazandaran- Behshahr** | **53.581** | **36.675** |  | **This study** |
| **KR075793** | ***S. scrofa*** | **MA5** | **Iran** | **Mazandaran- Behshahr** | **53.534** | **36.683** |  | **This study** |
| **KR075794** | ***S. scrofa*** | **MA6** | **Iran** | **Mazandaran- Neka** | **53.308** | **36.670** |  | **This study** |
| **KR075795** | ***S. scrofa*** | **MA7** | **Iran** | **Mazandaran** | **53.336** | **36.333** |  | **This study** |
| **KR075796** | ***S. scrofa*** | **MA8** | **Iran** | **Mazandaran** | **53.506** | **36.231** |  | **This study** |
| **KR075797** | ***S. scrofa*** | **MA9** | **Iran** | **Mazandaran- Kiasar** | **53.537** | **36.224** |  | **This study** |
| **KR075798** | ***S. scrofa*** | **MA10** | **Iran** | **Mazandaran- Kiasar** | **53.552** | **36.232** |  | **This study** |
| **KR075799** | ***S. scrofa*** | **MA11** | **Iran** | **Mazandaran- Neka** | **53.268** | **36.658** |  | **This study** |
| **KR075800** | ***S. scrofa*** | **GO1** | **Iran** | **Golestan- Gorgan** | **54.084** | **37.003** |  | **This study** |
| **KR075801** | ***S. scrofa*** | **GO2** | **Iran** | **Golestan- Gorgan** | **55.594** | **37.419** |  | **This study** |
| **KR075803** | ***S. scrofa*** | **GO3** | **Iran** | **Golestan- Gorgan** | **55.477** | **37.284** |  | **This study** |
| **KR075804** | ***S. scrofa*** | **GO4** | **Iran** | **Golestan- Gorgan** | **55.882** | **37.318** |  | **This study** |
| **KR075806** | ***S. scrofa*** | **GO5** | **Iran** | **Golestan- Gorgan** | **54.409** | **36.727** |  | **This study** |
| **KR075802** | ***S. scrofa*** | **GO6** | **Iran** | **Golestan- Gorgan** | **55.535** | **37.470** |  | **This study** |
| **KR075807** | ***S. scrofa*** | **KHSH1** | **Iran** | **Khorasan Shomali- Shirvan** | **57.955** | **37.428** |  | **This study** |
| **KR075809** | ***S. scrofa*** | **KHR1** | **Iran** | **Khorasan Razavi- Mashhad** | **59.208** | **36.446** |  | **This study** |
| **KR075810** | ***S. scrofa*** | **KHR2** | **Iran** | **Khorasan Razavi- Mashhad** | **59.201** | **36.428** |  | **This study** |
| **KR075811** | ***S. scrofa*** | **KHR3** | **Iran** | **Khorasan Razavi- Sarakhs Road** | **61.179** | **36.556** |  | **This study** |
| **KR075812** | ***S. scrofa*** | **KHR4** | **Iran** | **Khorasan Razavi- Daregaz** | **59.145** | **37.475** |  | **This study** |
| **KR075813** | ***S. scrofa*** | **KHR5** | **Iran** | **Khorasan Razavi- Daregaz** | **59.103** | **37.479** |  | **This study** |
| **KR075814** | ***S. scrofa*** | **KHR6** | **Iran** | **Khorasan Razavi- Quchan** | **58.723** | **37.181** |  | **This study** |
| **KR075808** | ***S. scrofa*** | **KHR7** | **Iran** | **Khorasan Razavi- Quchan** | **58.552** | **37.157** |  | **This study** |
| **KR075815** | ***S. scrofa*** | **KHR8** | **Iran** | **Khorasan Razavi- Quchan** | **58.294** | **37.257** |  | **This study** |
| **KR075816** | ***S. scrofa*** | **KHR9** | **Iran** | **Khorasan Razavi- Quchan** | **58.480** | **37.445** |  | **This study** |
| **KR075817** | ***S. scrofa*** | **KHR10** | **Iran** | **Khorasan Razavi- Quchan** | **58.208** | **37.501** |  | **This study** |
| **KR075818** | ***S. scrofa*** | **KHR11** | **Iran** | **Khorasan Razavi- Quchan** | **58.459** | **37.061** |  | **This study** |
| **KR075805** | ***S. scrofa*** | **KHR12** | **Iran** | **Khorasan Razavi- Chenaran** | **56.078** | **36.640** |  | **This study** |
| **AY884725** | ***S. scrofa*** | **GL254** | **Iran** |  |  |  | **NE1** | **[1]** |
| **DQ872947** | ***S. scrofa*** | **GL781** | **Iran** | **Kermanshah** |  |  | **NE1** | **[2]** |
| **DQ872969** | ***S. scrofa*** | **GL952** | **Sudan** |  |  |  | **NE1** | **[2]** |
| **AY884723** | ***S. scrofa*** | **GL252** | **Italy** |  |  |  | **Italian** | **[1]** |
| **AY884720** | ***S. scrofa*** | **GL248** | **Italy** |  |  |  | **Italian** | **[1]** |
| **AY884721** | ***S. scrofa*** | **GL249** | **Italy** |  |  |  | **Italian** | **[1]** |
| **AY884710** | ***S. scrofa*** | **GL236** | **Armenia** |  |  |  | **NE2** | **[1]** |
| **AY884727** | ***S. scrofa*** | **GL271** | **Armenia** |  |  |  | **NE2** | **[1]** |
| **AY884638** | ***S. scrofa*** | **GL77** | **Iran** |  |  |  | **Asiatic** | **[1]** |
| **AY884694** | ***S. scrofa*** | **GL194** | **Armenia** |  |  |  | **NE2** | **[1]** |
| **AY463065** | ***S. scrofa*** | **UNR123** | **Germany** |  |  |  | **European** | **[3]** |
| **AY463069** | ***S. scrofa*** | **DL** | **Germany** |  |  |  | **European** | **[3]** |
| **AY884690** | ***S. scrofa*** | **GL190** | **Italy** |  |  |  | **Italian** | **[1]** |
| **AY884693** | ***S. scrofa*** | **GL193** | **Armenia** |  |  |  | **NE2** | **[1]** |
| **AY884619** | ***S. scrofa*** | **GL55** | **Turkey** |  |  |  | **NE2** | **[1]** |
| **DQ872962** | ***S. scrofa*** | **GL912** | **Iran** |  |  |  | **NE2** | **[2]** |
| **DQ872958** | ***S. scrofa*** | **GL793** | **Iran** | **Sistan and Baluchistan, Zabol** |  |  | **NE2** | **[2]** |
| **DQ872966** | ***S. scrofa*** | **GL940** | **Turkey** | **Smyrna(Izmir)** |  |  | **NE2** | **[2]** |
| **DQ872940** | ***S. scrofa*** | **GL754** | **Turkey** | **Mersin (ifel) Tarsus Forest** |  |  | **NE2** | **[2]** |
| **DQ872938** | ***S. scrofa*** | **GL752** | **Iran** | **Fars, Yasuj, 10.9 mi SW** |  |  | **NE2** | **[2]** |
| **DQ872951** | ***S. scrofa*** | **GL786** | **Iran** | **Kermanshah** |  |  | **Asiatic** | **[2]** |
| **DQ872953** | ***S. scrofa*** | **GL788** | **Iran** | **Maku** |  |  | **Asiatic** | **[2]** |
| **DQ872957** | ***S. scrofa*** | **GL792** | **Iran** | **Sistan and Baluchistan, Zabol** |  |  | **Asiatic** | **[2]** |
| **DQ872980** | ***S. scrofa*** | **GL1009** | **Turkmenistan** |  |  |  | **Asiatic** | **[2]** |
| **DQ496829** | ***S. scrofa*** | **GL285** | **France** |  |  |  | **Asiatic** | **[1]** |
| **JQ668032** | ***S. scrofa*** |  | **Chili** |  |  |  | **European** | **GenBank** |
| **JQ273209** | ***S. scrofa*** | **SSDU23M06** |  |  |  |  | **European** | **[4]** |
| **JX894183** | ***S. scrofa*** | **WBTR514** | **Turkey** |  |  |  | **European** | **[5]** |
| **JX894161** | ***S. scrofa*** | **TK126** | **Turkey** |  |  |  | **NE2** | **[5]** |
| **JX894186** | ***S. scrofa*** | **WBTR517** | **Turkey** |  |  |  | **NE2** | **[5]** |
| **HM010482** | ***S. scrofa*** | **1082** | **Russia** |  |  |  | **Asiatic** | **[6]** |
| **EF545584** | ***S. scrofa*** | **Wbvnm 07** | **Vietname** |  |  |  | **Asiatic** | **[7]** |
| **AM040639** | ***S. scrofa*** |  |  |  |  |  | **Asiatic** | **GenBank** |
| **FJ601401** | ***S. scrofa*** | **bd06bSHWT** | **China** | **Jiangsu, Haimen county** |  |  | **Asiatic** | **[8]** |
| **FJ601392** | ***S. scrofa*** | **Bd01bJL** | **China** | **Hubei, jianli county** |  |  | **Asiatic** | **[8]** |
| **NC008830** | ***Ph. africanus*** | **Outgroup** | **Africa** |  |  |  |  | **[7]** |

**References:**

1. Larson G, Dobney K, Albarella U, Fang M, Matisoo-Smith E, Robins J, et al. Worldwide phylogeography of wild boar reveals multiple centers of pig domestication. Science. 2005;307(March):1618–21.

2. Larson G, Albarella U, Dobney K, Rowley-Conwy P, Schibler J, Tresset A, et al. Ancient DNA, pig domestication, and the spread of the Neolithic into Europe. Proc Natl Acad Sci U S A. 2007;104(39):15276–81.

3. Gongora J, Fleming P, Spencer PBS, Mason R, Garkavenko O, Meyer JN, et al. Phylogenetic relationships of Australian and New Zealand feral pigs assessed by mitochondrial control region sequence and nuclear GPIP genotype. Mol Phylogenet Evol. 2004;33(2004):339–48.

4. Goedbloed DJ, Megens HJ, Van Hooft P, Herrero-Medrano JM, Lutz W, Alexandri P, et al. Genome-wide single nucleotide polymorphism analysis reveals recent genetic introgression from domestic pigs into Northwest European wild boar populations. Mol Ecol. 2013;22(January 2012):856–66.

5. Ottoni C, Girdland Flink L, Evin A, Geörg C, De Cupere B, Van Neer W, et al. Pig domestication and human-mediated dispersal in western Eurasia revealed through ancient DNA and geometric morphometrics. Mol Biol Evol. 2013;30(4):824–32.

6. Ramayo Y, Shemeret’Eva IN, Pérez-Enciso M. Mitochondrial DNA diversity in wild boar from the Primorsky Krai Region (East Russia). Anim Genet. 2011;42(1):96–9.

7. Wu GS, Yao Y-G, Qu KX, Ding ZL, Li H, Palanichamy MG, et al. Population phylogenomic analysis of mitochondrial DNA in wild boars and domestic pigs revealed multiple domestication events in East Asia. Genome Biol. 2007;8(11):R245.

8. Larson G, Liu R, Zhao X, Yuan J, Fuller D, Barton L, et al. Patterns of East Asian pig domestication, migration, and turnover revealed by modern and ancient DNA. Proc Natl Acad Sci U S A. 2010;107(17):7686–91.
